# Polyploidy linked with species richness but not diversification rates or niche breadth in Australian Pomaderreae (Rhamnaceae)

**DOI:** 10.1093/aob/mcae181

**Published:** 2024-10-23

**Authors:** Francis J Nge, Timothy A Hammer, Thais Vasconcelos, Ed Biffin, Jürgen Kellermann, Michelle Waycott

**Affiliations:** School of Biological Sciences, Faculty of Science, The University of Adelaide, Adelaide, SA 5000, Australia; National Herbarium of New South Wales, Botanic Gardens of Sydney, Mount Annan, NSW 2567, Australia; State Herbarium of South Australia, Botanic Gardens and State Herbarium, Adelaide, SA 5000, Australia; IRD – Institut de Recherche pour le Développement, Montpellier, BP 64501, France; School of Biological Sciences, Faculty of Science, The University of Adelaide, Adelaide, SA 5000, Australia; State Herbarium of South Australia, Botanic Gardens and State Herbarium, Adelaide, SA 5000, Australia; Department of Ecology and Evolutionary Biology, University of Michigan, Ann Arbor, MI 48109, USA; School of Biological Sciences, Faculty of Science, The University of Adelaide, Adelaide, SA 5000, Australia; State Herbarium of South Australia, Botanic Gardens and State Herbarium, Adelaide, SA 5000, Australia; School of Biological Sciences, Faculty of Science, The University of Adelaide, Adelaide, SA 5000, Australia; State Herbarium of South Australia, Botanic Gardens and State Herbarium, Adelaide, SA 5000, Australia; School of Biological Sciences, Faculty of Science, The University of Adelaide, Adelaide, SA 5000, Australia; State Herbarium of South Australia, Botanic Gardens and State Herbarium, Adelaide, SA 5000, Australia

**Keywords:** Australian flora, diversification, niche space, phylogenomics, polyploidy, Rhamnaceae, turnover, woody

## Abstract

**Background and Aims:**

Polyploidy is an important evolutionary driver for plants and has been linked with higher species richness and increases in diversification rate. These correlations between ploidy and plant radiations could be the result of polyploid lineages exploiting broader niche space and novel niches due to their enhanced adaptability. The evolution of ploidy and its link to plant diversification across the Australian continent is not well understood. Here, we focus on the ploidy evolution of the Australasian Rhamnaceae tribe Pomaderreae.

**Methods:**

We generated a densely sampled phylogeny (90 %, 215/240 species) of the tribe and used it to test for the evolution of ploidy. We obtained 30 orthologous nuclear loci per sample and dated the phylogeny using treePL. Ploidy estimates for each sequenced species were obtained using nQuire, based on phased sequence data. We used MiSSE to obtain tip diversification rates and tested for significant relationships between diversification rates and ploidy. We also assessed for relationships between ploidy level and niche breadth, using distributional records, species distributional modelling and WorldClim data.

**Key Results:**

Polyploidy is extensive across the tribe, with almost half (45 %) of species and the majority of genera exhibiting this trait. We found a significant positive relationship between polyploidy and genus size (i.e. species richness), but a non-significant positive relationship between polyploidy and diversification rates. Polyploidy did not result in significantly wider niche space occupancy for Pomaderreae; however, polyploidy did allow transitions into novel wetter niches. Spatially, eastern Australia is the diversification hotspot for Pomaderreae in contrast to the species hotspot of south-west Western Australia.

**Conclusions:**

The relationship between polyploidy and diversification is complex. Ancient polyploidization events likely played an important role in the diversification of species-rich genera. A lag time effect may explain the uncoupling of tip diversification rates and polyploidy of extant lineages. Further studies on other groups are required to validate these hypotheses.

## INTRODUCTION

Polyploidy is an important evolutionary driver for plants (Lewis, 2004; Adams and Wendel, 2005; Wood *et al.*, 2009; Jiao *et al.*, 2011; Soltis and Soltis, 2016; Clark and Donoghue, 2018). Polyploids are the result of whole-genome duplication (WGD) events leading to these lineages having multiple sets of chromosomes (Stebbins, 1971). Polyploidization has been shown to result in greater adaptability for lineages, leading to their successful colonization of novel niches and more extreme environments (Te Beest *et al.*, 2012; López‐Jurado *et al.*, 2019; Baniaga *et al.*, 2020; Maguilla *et al.*, 2021; Edgeloe *et al.*, 2022). Indeed, polyploids are more common at higher latitudes (Brochmann *et al.*, 2004; Rice *et al.*, 2019), higher elevations (Wang *et al.*, 2023), and in deserts (Rice *et al.*, 2019; Chase *et al.*, 2023).

Polyploids have been linked to increased diversification in plants compared with their diploid congeners (Gorelick and Olson, 2011; Levin and Soltis, 2018; Ren *et al.*, 2018; Han *et al.*, 2020; Román-Palacios *et al.*, 2020; Meudt *et al.*, 2021; Heslop-Harrison *et al.*, 2023). However, other studies contradict this finding and show that recent polyploids diversify at lower rates (Mayrose *et al.*, 2011; but see Soltis *et al.*, 2014*a*), thus transitions to polyploidy do not always lead to an increase in species diversification (Landis *et al.*, 2018). A lag time in the diversification of polyploid lineages after a WGD event has been suggested as an explanation for this discrepancy (Levin, 2020). An interplay between novel traits derived from polyploidization (Zenil‐Ferguson *et al.*, 2019; Anderson *et al.*, 2023), response to the environment (Bouchenak‐Khelladi *et al.*, 2015) and clade-specific adaptability (Rice *et al.*, 2019) may offer a more complete view on the effects of polyploidy in diversification.

As it is suggested that polyploids are more successful and adaptable in unstable environments (e.g. glaciated regions; Stebbins, 1971, 1984; Soltis *et al.*, 2014*b*), we would expect regions with stable environments to have fewer polyploids as a proportion of the total flora. Indeed, the Cape Floristic Region of South Africa – a hyperdiverse floristic region with high historical climate and geological stability – has an anomalously low frequency of polyploid lineages compared to global patterns (Cowling *et al.*, 2015; Oberlander *et al.*, 2016; Rice *et al.*, 2019). Similarly, polyploidy is less common in older plant lineages of the Hengduan and Qinghai–Tibet region (Nie *et al.*, 2005; Sun *et al.*, 2017; Wang *et al.*, 2017) compared with younger lineages from the Andes (Schmidt‐Lebuhn *et al.*, 2010; Luebert and Weigend, 2014; Morales‐Briones *et al.*, 2018) or from regions that have experienced recent historical climatic instability, such as the Arctic (Brochmann *et al.*, 2004; Rice *et al.*, 2019).

Interestingly, the southwestern biodiversity hotspot region of Western Australia (SWA) has a relatively high polyploidy estimate (49.3 %) based on Hopper (1979), despite being another region with high geological and climatic stability (Hopper and Gioia, 2004; Nge *et al.*, 2020). However, this ploidy estimate of the SWA flora was based on a low sample size (75 observations) and subsequent studies with additional data show that SWA indeed has fewer polyploids than might be expected based upon global trends (Rice *et al.*, 2019).

The Australian continent has several distinct biomes, including the monsoonal north, the expansive arid interior, SWA with a Mediterranean climate, the temperate/alpine regions of south-eastern and eastern Australia, and the wet tropics of north-eastern Australia (Department of the Environment, 2013). The formation of these biomes through time and associated diversification of different plant lineages has been reviewed by others (Crisp *et al.*, 2004; Byrne *et al.*, 2008, 2011; Bowman *et al.*, 2010; Crisp and Cook, 2013; Byrne and Murphy, 2020). Gondwanan floristic elements with deep evolutionary histories in Australia are a dominant feature in historically more stable regions, such as SWA, south-eastern and eastern Australia (Crisp and Cook, 2013). Out of these three regions, it is understood that SWA has experienced greater climatic stability, buffered from major climatic changes from the Eocene towards the present (Sniderman *et al.*, 2013; Nge *et al.*, 2020).

The Australian alpine flora is relatively young compared to other floristic elements of the continent, with the assembly of most alpine lineages since the Pliocene (<5 Ma) (Crisp and Cook, 2013) and most lineages having dispersed from other surrounding landmasses, especially from New Zealand (e.g. Lockhart *et al.*, 2001; Bleeker *et al.*, 2002; Chung *et al.*, 2005; Meudt and Simpson, 2006; Gussarova *et al.*, 2008). The Australian arid flora also assembled relatively recently, with most of the dominant lineages radiating from the Miocene towards the present in response to the aridification of the continent (Cabrera *et al.*, 2011; Crisp and Cook, 2013; Schmidt-Lebuhn and Smith, 2016; Hancock *et al.*, 2018; Hammer *et al.*, 2021). Both arid and alpine regions in Australia experienced environmental instability in the form of desert dune formation and glaciation respectively during the Pliocene and Pleistocene (Ollier, 1986; Colhoun and Fitzsimons, 1990; Fujioka *et al.*, 2005, 2009).

The findings from Rice *et al.* (2019) of higher polyploid frequency in the arid and alpine areas of Australia is consistent with the general trend that polyploids have an evolutionary advantage in colonizing novel, unstable and extreme environments. The global study of Rice *et al.* (2019), while comprehensive in scope, still suffers from sampling bias with data gaps concentrated in the southern hemisphere and tropics (Vasconcelos, 2023). Taxon-intensive phylogenetic sampling to look at the recent diversification dynamics of polyploids is required to address this gap (e.g. Han *et al.*, 2020; Elliott *et al.*, 2023; Wang *et al.*, 2023). The role of polyploidy in spurring biome transitions and diversification across Australia (i.e. transitions into new niches) is currently not well understood, with just one study suggesting that polyploidy enabled biome transitions for Lomandroideae (Asparagaceae; Gunn *et al.*, 2020). As polyploids are thought to more readily colonize novel and extreme niches, it could be argued that on the other hand successful colonization and establishment of polyploid lineages into more species-rich regions would be harder due to competition from incumbent lineages (Silvestro *et al.*, 2015; Pavón-Vázquez *et al.*, 2022). The relationship between species richness and diversification rates spatially is not well established. Available studies have either supported this relationship (Pérez‐Escobar *et al.*, 2017) or found that diversification rates are uncoupled from species richness (Maestri *et al.*, 2019; Tietje *et al.*, 2022). Settling this debate first would be crucial in addressing the previous argument of whether colonization of species-rich environments is rarer for polyploids compared with novel niches/environments (with few incumbent species).

Polyploids have been documented for numerous Australian arid plant lineages (Randell, 1970; Shepherd and Yan, 2003; Sampson and Byrne, 2012; Anderson *et al.*, 2017; Dodsworth *et al.*, 2020) as well as the alpine (Castelli *et al.*, 2017) and southern temperate Australian plant lineages (e.g. Stewart and Barlow, 1976; Rye, 1979; Stace *et al.*, 1997; Shan *et al.*, 2003; Holmes *et al.*, 2009; Waters *et al.*, 2010; James and McDougall, 2014; Reiter *et al.*, 2015; Wallace *et al.*, 2019).

The tribe Pomaderreae (Rhamnaceae) is a diverse clade with ten genera and ~240 species, consisting of shrubs or small trees distributed across Australia (Kellermann, 2020*a*), with 8 species of *Pomaderris* found in New Zealand (Walsh, 1992; Nge *et al.*, 2021). Most species in the tribe occur across mesic southern regions of Australia including SWA, with few species occurring in the central arid interior and northern monsoonal tropics (Ladiges *et al.*, 2005; Kellermann, 2006; Clowes *et al.*, 2022; Nge *et al.*, 2023). *Cryptandra* and *Stenanthemum* are the two genera in Pomaderreae that have their distributions extending into the arid interior of Australia. The phylogenomic study of Nge *et al.* (2023) showed that arid-adapted *Cryptandra* species do not all stem from a single radiation event, but rather multiple independent transition events from temperature regions of Australia to the arid interior during the Miocene and Pliocene. The ploidy levels of these species are so far unknown. It has been estimated that a high proportion of species from the genus *Pomaderris* in Australia are thought to be polyploid (46 %; 17 of 37 taxa sampled in Chen *et al*., 2019), with the majority of these found to be triploids, including species that produce viable seeds (Chen *et al.*, 2019). With over 70 species, *Pomaderris* is the largest genus in the tribe Pomaderreae. The genus has most of its species distributed across the south-eastern mesic region of Australia, with several species found in SWA and north-eastern Australia (Nge *et al.*, 2021). It is not known whether polyploid lineages in *Pomaderris* were the result of a single WGD and subsequent radiation, or if these lineages were the result of multiple independent polyploidization events. Whether polyploidy occurs in other Australian Rhamnaceae genera besides *Pomaderris* and how many WGDs occurred in this plant group is currently unknown.

Given their distribution across Australia and the presence of polyploids, Pomaderreae is an ideal system to test for the impact of polyploidy on diversification through space and time, niche space occupancy and breadth. We hypothesize that (Table 1):

**Table 1.** Hypotheses proposed for this study looking into ploidy evolution of Pomaderreae.

Hypothesis	Explanation	References in support	Reference against
H1: Fewer polyploids in climatically stable regions, such as south-west Western Australia (SWA)	Polyploids are more successful and adaptable in unstable environments	Stebbins (1971), Stebbins (1984), Rice *et al.* (2019)	Hopper (1979)
H2: Polyploidy is linked with higher species richness and diversification rates, and also correlated in space	Polyploids linked with higher speciation and diversification rates due to genetic adaptability	Gorelick and Olson (2011), Levin and Soltis (2018), Ren *et al.* (2018), Han *et al.* (2020)	Mayrose *et al.* (2011)
H3: Polyploids occupy larger niche space and novel niches	Due to enhanced adaptability	Otto and Whitton (2000), Te Beest *et al.* (2012), Karunarathne *et al.* (2018), Van de Peer *et al.* (2021)	
H4: Polyploidy as a labile trait for Pomaderreae	Re-diploidization and independent origins of ploidy have been well documented in plants	Otto and Whitton (2000), Gunn *et al.* (2020)

(H1) fewer lineages in SWA would be polyploids compared with other regions of Australia, consistent with the expectation that polyploidy is uncommon in stable regions;

(H2) genera with a higher proportion of polyploids would have more species and higher diversification rates, and that species richness and diversification hotspots would also be spatially correlated;

(H3) polyploids occupy larger niche space (Karunarathne *et al*., 2018) and novel niches (Otto and Whitton, 2000; Te Beest *et al*., 2012) due to their enhanced adaptability (Van de Peer *et al*., 2021);

(H4) polyploidy is a labile trait across Pomaderreae, as has been shown for other plant groups (Otto and Whitton, 2000; Gunn *et al*., 2020).

## MATERIALS AND METHODS

### Rhamnaceae sequence and sampling

We included 264 samples representing all ten genera and 90 % (215/240 species) of all extant species diversity within the tribe (Supplementary Data Table S1). Most of the *Pomaderris* and *Cryptandra* sequence data were sourced from our previous published works (Nge *et al.*, 2021, 2023), whereas the other samples were newly sequenced for this study (Supplementary Data Table S1). We adopted a hybrid capture approach and utilized the OzBaits kit (Waycott *et al.*, 2021) to obtain up to 100 nuclear loci, and DNA sequence library preparation and sequence post-processing protocols followed those from our previous studies for Rhamnaceae (see details in Nge *et al.*, 2021, 2023).

### Polyploid inference

There are relatively few estimates of genome size or specific determinations of ploidy karyotypically for Australian species of Pomaderreae based on data available in large-scale databases such as the C-values database from Kew (Bennett and Leitch, 2012) and PloiDB (Halabi *et al.*, 2023), and only three *Pomaderris* species had chromosome count information available from the Tropicos Index to Plant Chromosome Numbers (IPCN) (Goldblatt and Johnson, 1979). Instead, to estimate ploidy level for samples in our study, we used the nQuire software (Weiß *et al.*, 2018), which estimates ploidy based on allelic frequency ratios of biallelic single-nucleotide polymorphisms (SNPs) derived from paired sequence reads. This approach has been demonstrated to obtain reliable ploidy estimates for other plants (Viruel *et al.*, 2019). However, Viruel *et al.* (2019) found that ploidy estimates above tetraploidy were unreliable due to sequencing noise obscuring the ploidy signal (i.e. it is hard to untangle whether allelic frequencies <0.2 are due to ploidy or sequencing quality). For these reasons, we categorized the ploidy estimates to three specific levels: diploids; triploids; and tetraploids and those above these specific assignable ploidies (referred to as ×4+). The software applies three-factor validation to estimate ploidy level: (1) observation of histograms showing distribution of allele frequencies; (2) best fit between ideal and empirical histograms; and (3) lowest delta likelihood values after comparing experimental data against maximized log-likelihood of the free model. In addition, we also validated our nQuire results with published ploidy estimates of *Pomaderris* derived from chromosome counts and genome size (C-value) estimates (Chen *et al.*, 2019).

### Environmental data and analyses

We obtained occurrence records based on herbarium vouchered specimens sourced from the Australasian Virtual Herbarium (http://avh.ala.org.au; accessed July 2021). For species that were just recently described or re-circumscribed (Kellermann, 2020*b*; Kellermann *et al.*, 2022*a*, *b*), the occurrence records were sourced from voucher specimens included in these studies. Geo-coordinates from these records were cleaned with Google OpenRefine v.3.4 (https://openrefine.org). Cultivated, introduced and erroneous records (e.g. locations located in oceans or outside the known distributional range) were trimmed. Search terms ‘botanical gardens’, ‘cultivated’ and ‘planted’ were used to search for cultivated specimens. Occurrence records of infraspecific taxa (e.g. subspecies or varieties) were merged up to the species level.

For H1, the number of species occurring in SWA were quantified and compared with non-SWA species based on the cleaned occurrence records. The SWA region was delimited using the Interim Biogeographic Regionalisation of Australia (IBRA) regions following Cook *et al.* (2015) and Nge *et al.* (2020). A *χ*^2^ test was conducted to test for significance between ploidy and number of species occurring in SWA.

A distributional dataset of the length (km) and area (km^2^) of the distributions for each species was compiled by mapping all occurrence records in QGIS v.3.16 (http://qgis.org). The lengths of the distributions were found by using the Measure Line tool (ellipsoidal option) for the two most distant occurrences for each species, and area was calculated by using the Measure Area tool (ellipsoidal option) and drawing a polygon around all occurrences for each species (convex hull, based on the outer distributional points). Narrowly endemic species were given a minimum distribution length of 1 km and area of 1 km^2^.

An environmental dataset that included bioclimatic variables and elevation was created by using the 19 standard bioclimatic variables (i.e. BioClim 1–19) and the elevation variable from WorldClim v.2 based on current conditions (1970–2000) and at a resolution of 30 arc-seconds (~1 km^2^) (Fick and Hijmans, 2017). These data layers were overlaid onto the occurrence records in QGIS, and the plugin Point Sampling Tool v.0.5.3 was used to extract the values of each variable for each occurrence. This dataset was summarized by calculating the minimum (min), maximum (max), range (max–min) and midpoint ([min + max]/2) values for each taxon across all these variables. Midpoint was used here instead of mean or median to avoid the geographical sampling bias inherent in herbarium collections (e.g. many more records along roadsides or clustered near high-population areas).

The area and length variables along with the range values of WorldClim variables were used as proxies of niche breadth, with the assumption that taxa with larger ranges have greater niche breadth as they occupy a larger area that spans greater climatic space. The midpoint values of WorldClim variables were used as proxies for investigating different niche occupancy across taxa. To visualize niche breadth across the different ploidy categories, a scaled principal component analysis (PCA) was implemented for all WorldClim variables, BIO1–19, in R using the ggplot2 (Wickham, 2016) and ggfortify packages (Tang *et al.*, 2016). Niche breadth and degree of niche overlap between the different ploidy categories were assessed through the 95 % confidence ellipses from the PCA.

### Divergence time estimation

We constructed a maximum likelihood (ML) tree with our concatenated nuclear alignment using RAxML v.8.2.9, using the GTR + GAMMA substitution model (Abadi *et al.*, 2019). This RAxML tree was then used as the input tree for our divergence-dating analysis. A sensitivity phylogenetic analysis was conducted following Jantzen *et al.* (2022), with a diploid-only subset (inferred from nQuire) to assess whether the generic relationships are similar between the subset and original full dataset. These topologies are similar, hence providing confidence to our results in this study (Supplementary Data Fig. S1), and subsequent analyses (divergence time, diversification) were carried out with the full dataset (including polyploid taxa). Given the size of our dataset, we used a penalized likelihood approach implemented in treePL (Smith and O’Meara, 2012) to obtain divergence time estimates for Pomaderreae, which is computationally efficient compared with other molecular clock methods (e.g. Bayesian approach: BEAST; Bouckaert *et al.*, 2014). A subset of four fossil calibrations applied from other Rhamnaceae studies (Hauenschild *et al.*, 2018; Tian *et al.*, 2024) were included in the treePL analysis: (1) Crown of Rhamnaceae (80–100 Myr; min–max), (2) Crown of Rhamneae (68–79 Myr), (3) Crown of Ziziphoids, which includes Pomaderreae (64.8–79 Myr), and (4) crown of *Ceanothus* (28.4–64 Myr). The putative *Pomaderris* macrofossil from New Zealand (Campbell, 2002) was not included in the present study due to uncertain taxonomic affinity but inclusion had no appreciable effect on divergence time estimates in previous studies (Nge *et al.*, 2021). For our treePL analysis, the best smoothing value was determined by examining the output of the cross-validation analysis and selecting the smoothing value with the lowest *χ*^2^ score (Maurin, 2020).

### Diversification analyses

To estimate rates of diversification along the phylogeny of Pomaderreae, we used the Missing State Speciation and Extinction (MiSSE) method available in the R package hisse (Beaulieu and O’Meara, 2016; Vasconcelos *et al.*, 2022). MiSSE is an extension of the state speciation and extinction models that uses hidden Markov models to estimate areas in the tree that are in distinct diversification rate categories without the need to assign trait information to the tips *a priori*. We ran MiSSE by establishing a sampling fraction of 0.86, assuming that 86 % of the species richness described for the Pomaderreae is sampled in the tree, and a maximum number of 22 distinct rate categories based on the size of the tree, i.e. total number of tips divided by 10. That resulted in a list of 231 models with different combinations of rate categories to be fitted in the tree. Models were run in groups of five at a time using the function MiSSEGreedy until the delta AICc among models in parallel runs was <2 (see more details in Vasconcelos *et al.*, 2022). The list of models was then pruned for redundancy using the function PruneRedundantModels and models with a different number of rate classes were averaged according to their AIC weight to net diversification and turnover parameters at the tips of the tree. These so-called tip rates can then be used as the equivalent of a continuous trait in phylogenetic comparative analyses and to map potential for diversification in space (see below). Because we are analysing ploidy estimates derived from extant taxa, focusing on recent diversification rates by using the tip rate method is particularly interesting in the specific context of our study.

### Species richness and net diversification maps

To map and visualize net diversification of Pomaderreae across their geographical distribution in Australia, we modelled the distribution of each species using the Maxent algorithm in the R package dismo (Hijmans *et al.*, 2017). We first used functions of the R packages sp and raster (Bivand *et al.*, 2013; Hijmans *et al.*, 2015) to thin the distribution data of each species with three or more occurrence points to reduce the impact of overcollection in some areas, reducing the dataset to three occurrence points for each 1 × 1 degree grid cell. Then we created a convex hull including a buffer of 250 km around all distribution points for each species to be used as a background area. The 19 WorldClim 2 variables at a resolution of 10 min (Fick and Hijmans, 2017) were cropped for the background area of each species, and a collinearity test was run to highly correlated variables using the function vifcor of the R package usdm (Naimi *et al.*, 2014) with a threshold of 0.8. The remaining variables for each individual species were used as predictors in each species distribution model analysis. We then created a set of randomly generated background points within the study area of each species, with an expansion factor of 1.25 to ensure enough points for model training and evaluation. The occurrence data were split into training and testing sets using *k*-fold cross-validation (*k* = 2). One fold was used for training the model, and the other fold was used for testing. Background points were similarly split into training and testing sets. Each resulting distribution model was then projected and transformed into a binary layer (i.e. presence = 1 and absence = 0) by using the 10th percentile training presence method to determine a binarization threshold specific for each species. For species with fewer than three occurrence points, or for species where fewer than three points remained after the thinning procedure, distribution was inferred based on a circle of 25 km radius around each occurrence point. Species richness maps were generated by overlapping and calculating the sum of all binary layers of all species. The number of presence points, final list of climatic predictors, binarization threshold and resulting AUC for the modelling of each species are available in Supplementary Data Table S2.

Tip net diversification rates for each species were assigned to the distribution layer of the corresponding species to calculate the mean net diversification rate per grid cell. Finally, to highlight areas where net diversification rates are particularly high or low given the local species richness, and because local species richness and rates of net diversification might be correlated, we performed a linear regression between species richness and mean net diversification layers and mapped the geographical distribution of the residual values. Positive residual values correspond to areas where net diversification is particularly high and negative values correspond to areas where net diversification is particularly low given the species richness. Scripts to replicate distribution and diversification analyses can be found in https://github.com/tncvasconcelos/rhamnaceae_ploidy.

### Ploidy-dependent diversification and niche breadth

To test for H2, we conducted Spearman rank correlation tests between genus size (i.e. total number of species per genus) and proportion of species in each genus with diploids, triploids, and tetraploids + (hereafter, polyploids). For H2, we expect genera with a greater proportion of polyploids will have a higher number of species. Genus size was log-transformed prior to analysis. To account for potential denominator-driven statistical bias (from using the proportion of polyploids per genus), we conducted a different statistical analysis with the total number of polyploids per genus instead of proportional values. A Poisson regression was performed in R to examine the statistical relationship between the total number of species and the number of polyploid species (triploid and tetraploid +) per genus. We also used the phylANOVA function (phylogenetic ANOVA) in the R package phytools (Revell, 2012) to test for significant associations between speciation, net diversification and turnover rates derived from MiSSE and ploidy level while accounting for phylogenetic relationships (H2). For ploidy level, we tested two categorization regimes: (1) diploid, triploid, tetraploid + (hereafter 3-PLOID); and (2) binary – diploid and polyploid (with triploids included, hereafter 2-PLOID).

To test for H3, we also applied phylogenetic ANOVA with ploidy as the independent variable (both 3-PLOID and 2-PLOID) and all 19 WorldClim variables, elevation (m), area (km^2^) and length (km) as dependent variables. All these variables were log-transformed prior to analysis, with all analyses conducted in R v.3.5.0 (R Core Team, 2016). The results were visualized using violin plots created with the geom_violin function from the ggplot2 package (Wickham, 2016) in R.

Lastly, to test for H4 and assess for phylogenetic signal and trait lability, Fritz’s *D*-statistic (Fritz and Purvis, 2010) was calculated for ploidy as a binary trait (2-PLOID) using the caper R package (Orme *et al.*, 2013). A *D*-statistic value of 0 indicates the presence of phylogenetic signal corresponding to Brownian motion evolution, whereas a value of 1 indicates a complete absence of phylogenetic signal (i.e. the traits are randomly distributed across the phylogeny). For 3-PLOID, we tested for phylogenetic signal using Blomberg’s *K* (Blomberg *et al.*, 2003) and Pagel’s *λ* (Pagel, 1999) via the phylosig function in phytools. For Blomberg’s *K*, we specified the phylosig function to calculate the *K* value based on 1000 replicates of random assignment of diversification rate values to species, in order to assess for significance. We also tested whether rates of polyploidization are higher in younger diversifying clades, indicative of selective pressure for polyploidy coinciding with the exploration of novel niche space. We obtained the divergence age of each polyploid taxon from our treePL phylogeny and conducted phylogenetic ANOVA analyses in R for both 2-PLOID and 3-PLOID, with the assumption that polyploidization occurred since the branching event of each taxon with its sister clade.

## RESULTS

### Phylogenetic tree

We obtained ~30 orthologous nuclear loci (Supplementary Data Table S3), with a total alignment length of 35 560 bp covering 215 taxa. The final dataset comprises 199 taxa, which were included in (1) the dated phylogeny, (2) the ploidy analysis, and (3) geographical range size estimates; i.e. 16 taxa were excluded as we could not estimate a meaningful geographical range size for them (narrow endemics occurring in just one location each). Our RAxML tree resolved all ten genera in Pomaderreae as monophyletic, with high support (Supplementary Data Fig. S1). There were 11 species of *Pomaderris* included in our study that were also used in Chen *et al.* (2019). Of these, 9/11 (82 %) had comparable ploidy estimates between the two studies using different methods, providing additional confidence of these results (Supplementary Data Table S4). Seven of the 11 taxa were based on the tissue obtained from the same individual for the previous (Chen *et al.*, 2019) and this study. The two taxa with conflicting ploidy estimates (*Pomaderris bodalla* and *P. obcordata*) were found to be diploids by Chen *et al.* (2019) but triploids using nQuire in this study. The *P. obcordata* sample had poor sequence coverage (~600 k reads) and hence the conflicting ploidy estimates may result from low data quality. The conflicting ploidy estimate for *P. bodalla* is unexpected, new chromosome counts and re-sequencing may be required to investigate this anomaly.

### Spatial diversification and ploidy in Pomaderreae

We show that, across Pomaderreae, diversification and species hotspots were uncoupled, as high diversification areas in eastern Australia were driven by the recent radiation of *Pomaderris*, thus rejecting H2. Indeed, eastern Australia was no longer a hotspot for species richness once *Pomaderris* was excluded from the dataset (Supplementary Data Fig. S2).

**Fig. 1.**
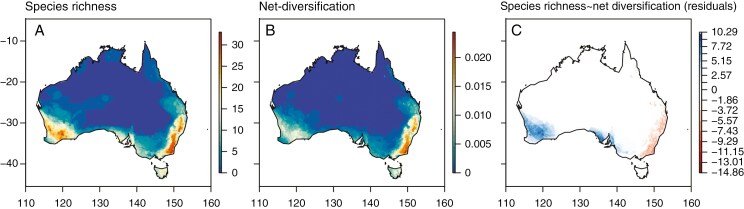
Maps derived from cleaned herbarium occurrence data and modelled species distributions (Maxent) showing (A) species richness (high–low; red–blue), (B) net diversification rates (high–low; red–blue) and (C) residuals between the two for Pomaderreae (Rhamnaceae) across Australia. Left *y*-axis and *x*-axes respectively show latitude and longitude coordinates.

Species richness hotspots for Pomaderreae are found in both SWA and eastern Australia (Fig. 1). Within SWA, species hotspots are found across the Wheatbelt, northern and southern sandplain Interim Biogeographic Regionalisation of Australia (IBRA) regions, all with prominent heath–shrub kwongan vegetation. Conversely, the high-precipitation south-west corner of SWA is relatively depauperate in Pomaderreae species. In eastern Australia, the Great Dividing Range has the highest levels of species richness for Pomaderreae, followed by the southern peninsulas of South Australia (Eyre, Yorke and Fleurieu peninsulas).

**Fig. 2.**
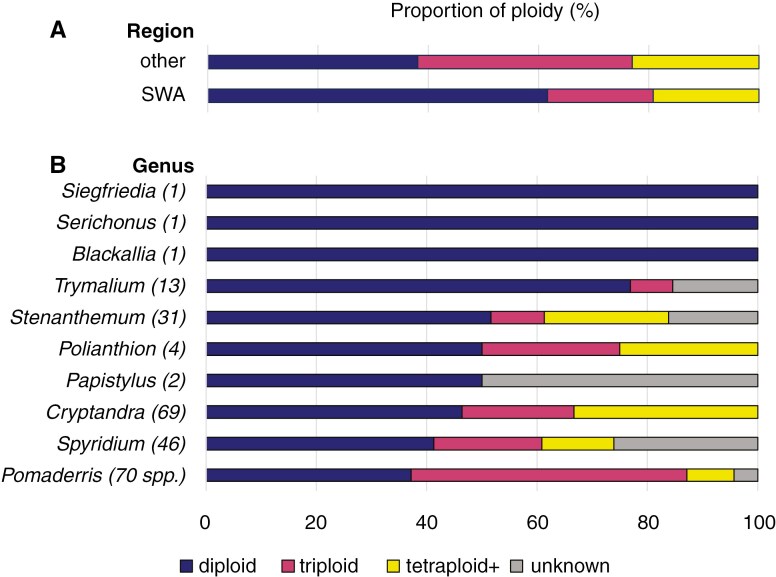
Proportion of different ploidy levels for Pomaderreae in (A) south-west Western Australia (SWA) and other regions across Australia, and (B) across different genera, numbers in brackets indicate species diversity for each genus. Ploidy levels were estimated using nQuire based on sequence data.

We found support for H1, where SWA has significantly fewer polyploids (38 %; 28/73) than other regions (62 %; 78/126) based on the *χ*^2^ test (*P* < 0.01; Fig. 2). This pattern is consistent across ploidy levels – triploidy and tetraploidy + (Fig. 2).

### Extensive polyploidy linked to species richness in Pomaderreae

**Fig. 3.**
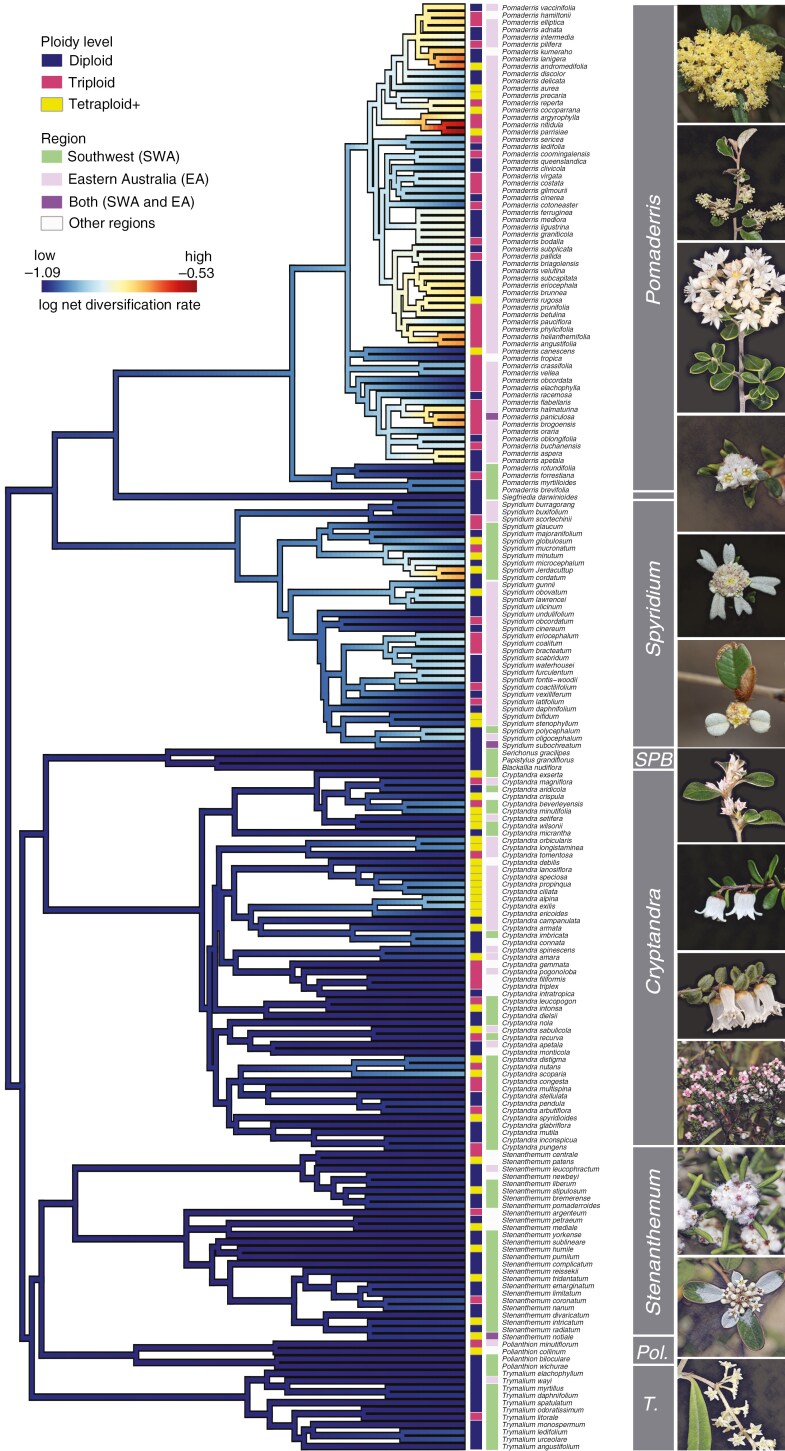
Dated treePL phylogeny showing tip-specific diversification rates estimated from MiSSE; heat colours show low–high (blue–red) diversification rates. Ploidy level and geographic range of each taxon are also shown with different colour schemes. The ‘SPB’ clade includes *Serichonus*, *Papistylus* and *Blackallia*. ‘*Pol*.’ represents *Polianthion*, and ‘*T*.’ represents *Trymalium* in the genus-delimiting box. All photographs taken by Francis J. Nge (unless stated otherwise). Top to bottom: *Pomaderris lanigera*, *Pomaderris paniculosa, Pomaderris brevifolia* (photo by K.R.Thiele), Spyridium ericoides*, *Spyridium bifidum*, *Spyridium thymifolium*, *Serichonus gracilipes* (photo by K.R.Thiele), *Cryptandra amara*, *Cryptandra arbutiflora* var. *arbutiflora* (photo by K.R.Thiele), *Cryptandra myriantha* (South Australian form), *Stenanthemum humile*, *Stenanthemum pomaderroides*, *Trymalium wayi.*

We found evidence for the presence of polyploids across the majority of genera in Pomaderreae (7/10; Figs 2 and 3). Of these, *Pomaderris* had the greatest number and proportion of its species as triploids (50 %, 35/70 species). *Cryptandra* had the greatest number and proportion of polyploids (tetraploid +; 33 %, 23/69 species). All three monotypic genera (*Siegfriedia*, *Serichonus*, *Blackallia*) from SWA are diploids. We found significant support for H2, as diploidy is negatively correlated with genus size (𝜌 = −0.88, *P* < 0.001; Supplementary Data Table S5). In addition, both triploidy and tetraploidy + are positively associated with genus size (𝜌 = 0.85 and 0.68 respectively, *P* < 0.01; Supplementary Data Table S5). Polyploidy in general is also positively associated with genus size based on our Poisson regression analysis (*P* < 0.001; Supplementary Data Table S6).

**Fig. 4.**
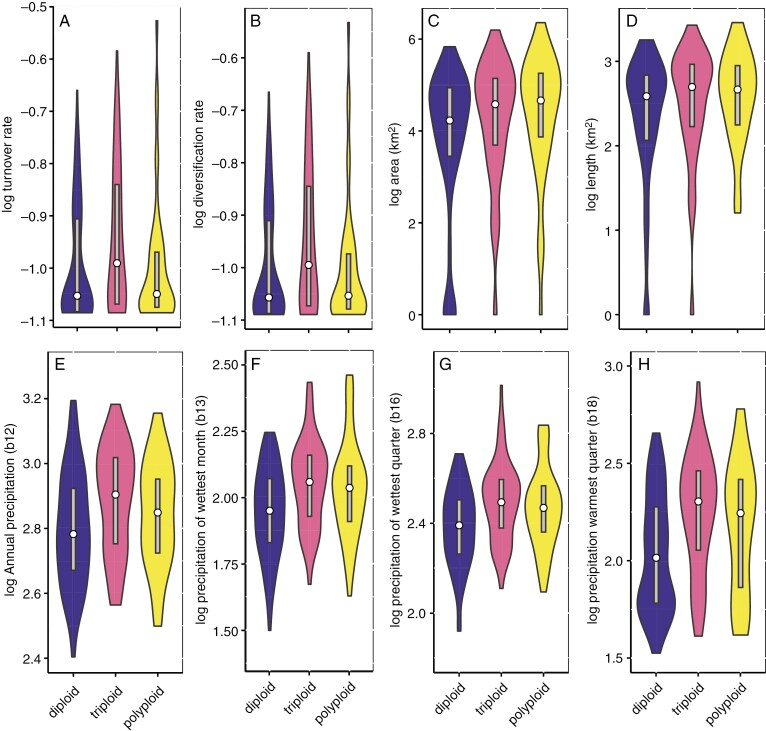
Violin plots of ploidy with different diversification metrics and WorldClim environmental variables (proxy for niche space). (A) Turnover rate, (B) diversification rate, (C) distributional range size, (D) distributional length, (E) annual precipitation, (F) precipitation of wettest month, (G) precipitation of wettest quarter, (H) precipitation of warmest quarter. Only WorldClim variables having significant associations with ploidy are shown. Values represent midpoints. The width of each violin plot indicates density of values, and the white circles the value of the median. Grey bars represent values within a factor of 1.5 of the upper and lower quartiles.

The net diversification, speciation and turnover rates were higher for triploids than diploids and polyploids, primarily driven by *Pomaderris* (Fig. 3, Supplementary Data Fig. 3). However, these differences were not significant once phylogenetic relatedness was taken into account (phylANOVA; Fig. 4), hence rejecting H2.

### Polyploidy, niche breadth, niche occupancy and phylogenetic signal

Polyploids have a greater distributional range (niche breadth) compared with diploids; however, these differences are non-significant, therefore rejecting H3 (Fig. 4C, Table 2). Similar findings were noted for the range values of our WorldClim dataset (Table 2). We found that niche occupancy across different precipitation regimes was significantly different between diploids and polyploids (phylANOVA), across both 3-PLOID and 2-PLOID analyses, thus supporting H3 (Table 2). Polyploids (triploids and tetraploids +) occur in wetter areas with more annual precipitation (WorldClim B12) and during the wettest months (B13) and wettest quarter (B16), whereas more diploids occur in areas with a Mediterranean climate, i.e. SWA and South Australia (indicated by low precipitation of warmest quarter, WorldClim B18) (Fig. 4). Triploids occur in a significantly higher elevation range than diploids, but these differences became non-significant when phylogenetic relatedness (phylANOVA) was taken into account (Table 2, Supplementary Data Fig. S4). Overall, there was substantial overlap in niche breadth across WorldClim variables (BIO1–19) for taxa across all ploidy levels (PCA, Supplementary Data Fig. S5).

**Table 2.** Summary of correlations between diversification metrics and bioclimatic WorldClim variables with polyploidy (3-PLOID and 2-PLOID) from phylANOVA analyses, accounting for phylogenetic relatedness. Significant relationships are highlighted in bold and * and ** represent significant *P* values of <0.01 and <0.001 respectively. All variables were log-transformed prior to analyses.

	3-PLOID (diploid, triploid, tetraploid +)			2-PLOID (diploid, polyploid)		
Variable	Sum of squares	Residual	*P-*value	Sum of squares	Residual	*P-*value
Net diversification rate	0.11	2.91	0.25	0.05	2.99	0.36
Turnover rate	0.11	2.93	0.25	0.05	2.97	0.40
Distributional area (km^2^)	11.43	442.26	0.39	11.25	442.45	0.27
Distributional length (km^2^)	1.99	100.36	0.54	1.89	100.47	0.33
Elevation (m)	0.03	17.18	0.95	0.00	17.21	0.93
BIO1 = annual mean temperature	0.01	1.08	0.82	0.00	1.09	0.80
BIO2 = mean diurnal range [mean of monthly (max. temp. − min. temp.)]	0.03	0.72	0.20	0.02	0.73	0.25
BIO3 = isothermality (BIO2/BIO7) (× 100)	0.00	0.12	0.86	0.00	0.12	0.77
BIO4 = temperature seasonality (standard deviation × 100)	0.06	0.91	0.07	0.03	0.94	0.15
BIO5 = maximum temperature of warmest month	0.02	0.58	0.26	0.01	0.59	0.41
BIO6 = minimum temperature of coldest month						
BIO7 = temperature annual range (BIO5 − BIO6)	0.04	0.66	0.11	0.02	0.68	0.21
BIO8 = mean temperature of wettest quarter	0.01	3.01	0.88	0.01	3.02	0.71
BIO9 = mean temperature of driest quarter	0.12	2.33	0.14	0.07	2.40	0.24
BIO10 = mean temperature of warmest quarter	0.02	0.72	0.54	0.00	0.73	0.61
BIO11 = mean temperature of coldest quarter	0.00	2.31	0.96	0.00	2.32	0.93
**BIO12 = annual precipitation**	0.52	5.67	0.012*	0.44	5.75	0.009**
**BIO13 = precipitation of wettest month**	0.59	5.87	0.008**	0.55	5.90	0.002**
BIO14 = precipitation of driest month	0.50	17.20	0.38	0.25	17.25	0.43
BIO15 = precipitation seasonality (coefficient of variation)	0.04	6.70	0.85	0.00	6.73	0.89
**BIO16 = precipitation of wettest quarter**	0.54	5.99	0.018*	0.50	6.02	0.008**
BIO17 = precipitation of driest quarter	0.39	14.49	0.40	0.21	14.67	0.40
**BIO18 = precipitation of warmest quarter**	1.82	18.15	0.007**	1.66	18.32	0.003**
BIO19 = precipitation of coldest quarter	0.09	8.75	0.76	0.02	8.81	0.78

We obtained a *D*-statistic value of 0.64 for 2-PLOID that is significantly different from Brownian phylogenetic structure, thus supporting H4 (*P* < 0.01; Supplementary Data Table S7). Similarly, no significant phylogenetic signal for 3-PLOID was detected across both Blomberg’s *K* and Pagel’s *λ*, indicating that ploidy as a trait is labile across Pomaderreae and not phylogenetically conserved (Supplementary Data Table S8). We found that younger Pomaderreae lineages do not have more polyploids (as a proxy for rates of polyploidization events) than older lineages, based on phylogenetic ANOVA analyses (Supplementary Data Table S9).

## DISCUSSION

### Polyploid frequency

This study presents the first densely sampled, well resolved phylogeny for Pomaderreae (Rhamnaceae) with quantified ploidy across the entire tribe to look at macroevolutionary dynamics at a broad scale. We show that polyploidy is widespread and extensive, not just limited to *Pomaderris*, but also found throughout the tribe. We found that almost half (44.1 %; 105/238 species) of the taxa in Pomaderreae and the majority of genera (70 %; 7/10) exhibit polyploidy. This estimate is unlikely to change significantly for Pomaderreae as we have sampled almost all known species within the tribe (91 % sampling; 23 species not included here). Interestingly, this estimate is similar to that of Chen *et al.* (2019), who, using a different methodological approach to ours, arrived at an estimate of 46 % based on just 30 *Pomaderris* species. Congruence between determining ploidy from sequence data using nQuire and from traditional flow cytometry studies has also been shown in other studies of different plant groups (Viruel *et al.*, 2019; Jantzen *et al.*, 2022). The few conflicts detected in our side-by-side comparison of the same samples evaluated by the two methods should be investigated further, to determine whether this was due to intraspecific polyploidy or from the nature of sequence data obscuring true signal, e.g. from low sequence coverage, the level of heterozygosity across loci, or the presence of paralogs. In our case, the last option seems less likely given that we had specifically filtered out all potential paralogous genes and only used 30 orthologous loci. Nevertheless, we show here that nQuire provides a promising avenue to scale up efforts in estimating ploidy across a wide range of plant groups with available sequence data, provided appropriate verification steps are taken with available chromosome-count studies.

The high rate of polyploidy in Pomaderreae is intriguing as all species in the tribe are woody shrubs or small trees. In the global study of Rice *et al.* (2019) comprising 2000+ species, the average polyploidy frequency was 33.5 % for herbs and 22 % for woody species; both estimates are substantially less than the estimate we obtained for Pomaderreae. Polyploidy has been observed to be more common generally in herbaceous than woody plants (Stebbins Jr, 1938; Levin and Wilson, 1976; Zenil‐Ferguson *et al.*, 2019), again different from our study. Few group-specific studies on polyploidy in woody plants are available, as a large proportion of polyploidy studies have had a focus on herbaceous lineages. A study on another woody plant genus, European *Salix* (Salicaceae), had a similar polyploidy estimate (21.2 %; 7/33 species) to the global average from Rice *et al.* (2019). Based on these findings, we surmise that Pomaderreae has an unusually high proportion of polyploid taxa compared with other woody plants. To date, only one other woody genus, *Polylepis* (Rosaceae), has a similarly high polyploidy rate (54.5 %; 18/33 species) to that of Pomaderreae (Schmidt‐Lebuhn *et al.*, 2010). Interestingly both Rosaceae and Rhamnaceae belong to the same order (Rosales; Liu *et al.*, 2023). Although we acknowledge that there is a paucity of ploidy data for tropical woody plants (Vasconcelos, 2023), comparisons such as those listed above are likely limited by the gap in data availability for a wider range of groups, potentially reflecting sampling bias. More detailed studies on other woody plant lineages will be required to better understand the distribution and traits associated with woody plant groups that have higher polyploidy frequencies than others.

We also document an unexpectedly high frequency of triploids in the Pomaderreae (26.4 %; 63/238 species). The largest genus in Pomaderreae (*Pomaderris*) was observed to have the greatest proportion of triploids (50 %; 35/70 species), in line with previous studies based on flow cytometry (Chen *et al.*, 2019). The evolutionary and ecological mechanisms leading to this high frequency are likely to be complex and relate to the steps involved in their origins. For example, several of the triploid species in Pomaderreae are known to be rare, such as *Pomaderris pallida* and *P. reperta*, with *P. pallida* shown to produce very little viable seed (Chen *et al.*, 2019). Triploids (as reproductive adults) are often documented with reduced fertility, genomic instability, and chromosome mis-pairing (Ramsey and Schemske, 1998; Comai, 2005; Wang *et al.*, 2010). Such genomic instability may lead to their relatively short-lived existence across deeper evolutionary timescales. Triploids are also often found in mixed-ploidy populations of species as discussed in the work of Husband (2004), now observed in numerous other plants, e.g. *Gingko biloba* (11 % [3/26 individuals]; Šmarda *et al.*, 2018). Our findings demonstrate that triploids have a higher turnover rate than diploids or other polyploids in the Pomaderreae as expected due to genomic instability. Thus, while polyploidization can confer potential evolutionary advantages of greater adaptability and heterosis, polyploid lineages would also need to overcome genomic instability and other disadvantages associated with WGD events (reviewed in Comai, 2005). It is also worth noting that differences in turnover rates for triploids versus lineages with other ploidy levels were not significant once phylogenetic relatedness was factored in, perhaps because many of the triploid species are closely related within *Pomaderris*, an interesting finding in itself. A comprehensive survey is warranted to test whether the findings of these studies can be expanded to the wider tribe, with important conservation implications, particularly if intervention-based restoration works were planned.

### Polyploidy, species richness and diversification

For Pomaderreae, we found a significant positive relationship between polyploidy and genus size, with larger genera having a higher proportion of polyploids, consistent with studies on other plant groups (Petit and Thompson, 1999; Otto and Whitton, 2000; Vamosi and Dickinson, 2006). Indeed, this pattern holds true even when accounting for phylogenetic sister pairs (*Cryptandra–Blackallia*, *Papistylus*, *Serichonus*, *Trymalium–Polianthion*, *Stenanthemum*, and *Pomaderris*–*Siegfriedia*). In the case of diversification tip rates across the entire tribe, a positive relationship with polyploidy was also noted, albeit non-significant. This finding shows that polyploidy does not always lead to increases in diversification rates (i.e. the speed at which species radiate), in agreement with some studies (Mayrose *et al.*, 2011; Arrigo and Barker, 2012; Scarpino *et al.*, 2014; Zhan *et al.*, 2014) but nevertheless in contrast to others showing the opposite (e.g. Meudt *et al.*, 2015; Han *et al.*, 2020; Moeglein *et al.*, 2020; Zhan *et al.*, 2021). There may also be a lag time since polyploidization and subsequent diversification (Schranz *et al.*, 2012; Levin, 2020). In addition, we argue here that links between diversification and polyploidy may also be temporally dependent, with older WGD events driving the diversification of species-rich genera, but on the other hand tip diversification rates and polyploidy of extant species are uncoupled. It could be that genetic stabilization following a WGD event is required prior to subsequent diversification of lineages, due to the inherent nature of genomic instability of young polyploids (Mayer and Aguilera, 1990; Comai, 2005; Gonzalo, 2022). The amount of time required for such stabilization processes to occur over deeper timescales is not well known and is likely clade-specific (e.g. Huang *et al.*, 2016, 2019; Clarkson *et al.*, 2017; Ren *et al.*, 2018; Smith *et al.*, 2018; Walden *et al.*, 2020).

Our results based on tip diversification rates add to the debate on whether neo-polyploids are ‘evolutionary dead-ends’, as has been argued by Mayrose *et al.* (2011) and Arrigo and Barker (2012), but disputed by Soltis *et al.* (2014*a*). No significant association between diversification rate and polyploidy was demonstrated in our study, countering Soltis *et al.* (2009). We found that Pomaderreae lineages exhibiting polyploidy did not have higher extinction rates either, thus also not fully supporting Mayrose *et al.* (2011). Though extinction rates through time are notoriously difficult to estimate from extant phylogenies (Rabosky, 2010), it could very well be that more polyploids have simply gone extinct and hence are not detected in the phylogeny.

The lack of significant correlation between diversification rates and polyploidy in Pomaderreae may be dependent on the scale of focus, as indicated by Zhan *et al.* (2014), where ordinal and family-level analyses showed non-significant associations in contrast to subfamilial analyses. Polyploidy has been inferred for other Rhamnaceae genera from other tribes, including *Colubrina*, *Rhamnus* and *Frangula* (Kew Plant DNA C-values Database: https://cvalues.science.kew.org/; accessed November 2021) and in this study based on sequence data (Supplementary Data Table S10). How extensive polyploidy is in non-Pomaderreae lineages and whether polyploidy is linked with diversification rates for these groups currently remains unknown. At least three WGD events have been documented for Rosales and hence it would not be surprising if extensive polyploidy is also found in other Rosales lineages besides Pomaderreae (Landis *et al.*, 2018). These WGDs may explain the paralleled successful colonization and *in situ* diversification across multiple temperate and Mediterranean biomes globally since the Oligocene for Rhamnaceae (Tian *et al.*, 2024).

Elevated species richness patterns are not always associated with higher diversification rates, indeed in many cases they are uncoupled (Pontarp and Wiens, 2017; Rabosky *et al.*, 2018; Igea and Tanentzap, 2020; Tietje *et al.*, 2022). Older clades or regions may have more time to accumulate more species instead of exhibiting higher diversification rates through shorter periods of time (Cook *et al.*, 2015; Andriananjamanantsoa *et al.*, 2016; Nge *et al.*, 2020). For Pomaderreae, a positive diversification rate shift has been inferred at the crown of *Pomaderris* (F.J.N., unpubl. res.) with a recent radiation in eastern Australia (Nge *et al.*, 2021). However, *Pomaderris* is not the only genus that exhibits polyploidy, indeed we show here that other species-rich genera within the tribe (*Cryptandra*, *Stenanthemum*, *Spyridium*) with older radiations in the Miocene also consist of many polyploid lineages. This uncoupling of diversification rates and species richness is not only seen across clades, but also spatially across Australia for Pomaderreae. We show that the diversification hotspot for Pomaderreae in eastern Australia was driven predominantly by one genus (*Pomaderris*) and that the SWA species hotspot did not exhibit elevated diversification rates. This uncoupling of spatial diversification and species richness hotspots has not been explicitly investigated in the literature, with few studies looking at diversification rate hotspots (e.g. Pérez‐Escobar *et al.*, 2017; Maestri *et al.*, 2019). The uncoupling also differs from other spatial diversification metrics, e.g. phylogenetic diversity and endemism, metrics that are more commonly used and usually correlate with centres of species richness (Tucker and Cadotte, 2013; Voskamp *et al.*, 2017; Xu *et al.*, 2021).

Our spatial results add to the growing literature highlighting the SWA region as a centre for plant diversity (Beard *et al.*, 2000; Hopper and Gioia, 2004; González-Orozco *et al.*, 2011; Prentice *et al.*, 2017) due to more time for species accumulation compared to other regions (Cardillo and Pratt, 2013; Sniderman *et al.*, 2013; Jabaily *et al.*, 2014; Cook *et al.*, 2015; Skeels and Cardillo, 2019; Nge *et al.*, 2022), instead of elevated diversification rates. The SWA region experienced relative climatic and tectonic stability (Hopper, 2009; Nge *et al.*, 2020) compared to other regions across Australia (Byrne *et al.*, 2008; Sniderman *et al.*, 2013), allowing the accumulation of hyperdiversity and retention of long-persisting lineages, e.g. species-poor endemic families: Cephalotaceae, Dasypogonaceae, Ecdeiocoleaceae and Emblingiaceae (Hopper and Gioia, 2004). We also show that SWA has significantly fewer polyploids than other regions for Pomaderreae, consistent with H1 and Oberlander *et al.* (2016) that stable regions have fewer polyploids. Indeed, the majority of Pomaderreae in SWA sampled for this study are diploids (62 %), similar to the study of Rice *et al.* (2019) but higher than that of Hopper (1979), which had a diploidy estimate of 50.7 % for the SWA flora. Lower levels of polyploidy in SWA compared with other less stable environments further support the view that polyploidy confers an evolutionary advantage during periods of environmental change linked with the opening of new niches (Baack, 2005; Oswald and Nuismer, 2011), e.g. during the Cretaceous–Palaeogene (K–Pg) mass extinction event (Fawcett *et al.*, 2009; Levin and Soltis, 2018). Or indeed, recurring episodes of population isolation and secondary contact may also lead to the formation of more polyploids in more unstable environments (Petit *et al.*, 1999; Hanzl *et al.*, 2014; Hülber *et al.*, 2015; Levin, 2019). This raises the question of whether, in the absence of large-scale environmental change, it would be evolutionarily advantageous for polyploids to undergo re-diploidization or face extinction. It is worth noting here that all of the monotypic SWA genera (*Siegfriedia*, *Serichonus*, *Blackallia*; i.e. species-poor lineages) are diploids, in stark contrast to other younger species-rich genera in Pomaderreae; hence the relationship between polyploidy, the timing of WGD events, and species richness warrants further investigation.

Phylogenetic uncertainty may affect these findings, but we stress here that a conservative approach was taken where out of 100 nuclear loci only 30 single-copy (orthologous) loci with no evidence of paralogy were used for phylogenetic reconstructions. Additional steps in the bioinformatic pipeline may be implemented to recover additional nuclear loci in future studies (Nauheimer *et al.*, 2021; Jackson *et al.*, 2023; Joyce *et al.*, 2024). Encouragingly, Naranjo *et al.* (2024) found that single-copy loci are reliable in inferring phylogenetic relationships even for polyploid taxa. Furthermore, our sensitivity phylogenetic analysis of diploid-only taxa and complete phylogeny with polyploids both recovered similar topologies. These findings along with our generally well-supported phylogeny suggest that phylogenetic uncertainty would not significantly affect our results.

### Polyploidy, niche breath and novel niche occupancy

Polyploidy in Pomaderreae did not result in significantly wider niche space occupancy per lineage. This suggests that greater adaptability as a result of polyploidization leads to niche divergence (usually into more extreme environments) from diploid congeners rather than an expansion of niche space (Ebersbach *et al.*, 2018; López‐Jurado *et al.*, 2019; Han *et al.*, 2020). For Pomaderreae, polyploidy allowed transitions into novel niche spaces in Australia with a wetter climate in eastern Australia. This result, i.e. that polyploids in Pomaderreae occupy wetter niches compared with diploids, is surprising, as it counters the findings of others. Stebbins Jr (1949) suggested that polyploids may be predisposed to colonize drier habitats. This pre-adaptation to drier, more extreme environments for polyploids has also been suggested for lineages in Australia (Gunn *et al.*, 2020), the Cape Floristic Region of South Africa (Bruyns *et al.*, 2019; Elliott *et al.*, 2023), the Iberian peninsula (Manzaneda *et al.*, 2012), and at a global scale (Hutang *et al.*, 2023). In contrast, only a few transitions into the arid biome of Australia were documented for Pomaderreae (F.J.N. *et al*., unpubl. res.), and none for the species-rich genus *Pomaderris* (Nge *et al.*, 2021). The Pomaderreae lineages that occur in the arid biome have thus far failed to radiate (e.g. *Stenanthemum centrale*, *Cryptandra imbricata–C. connata*, *C. monticola*, *C. crispula*, *C.* sp. Mukinbudin as part of *C. apetala sens. lat.*). In addition, lineages in Pomaderreae have suffered speciation declines towards the present, likely due to progressive aridification of the Australian continent (Nge *et al.*, 2023).

Phylogenetic niche conservatism and biome fidelity is a common phenomenon in the Australian flora (Crisp and Cook, 2013; Nge *et al.*, 2022), and one that applies to Pomaderreae as well. Another interesting point relates to the colonization of new habitats by several *Pomaderris* lineages, which have been shown to have dispersed independently several times from Australia to New Zealand in the Pliocene–Pleistocene (Nge *et al.*, 2021). All of the New Zealand *Pomaderris* species except for one (*P. kumeraho*) are polyploids, though they have failed to radiate (Dawson, 2000). This finding is at odds with the general trend where polyploids are shown to both diversify more readily in new environments (novel niche occupancy) and on islands (Meudt *et al.*, 2021). Why polyploid *Pomaderris* have failed to radiate in New Zealand is currently unknown but may be related to competition from incumbent lineages (e.g. Betancur‐R *et al.*, 2012; Pavón-Vázquez *et al.*, 2022), and perhaps a lag time of increased diversification after a polyploidization (WGD) event (Levin, 2020). Based on the findings above, we argue that, while polyploidization should result in greater adaptability, this does not always mean that polyploids will be successful in diversifying across more extreme environments. Links between polyploidy and niche diversification are likely group-specific, and dependent on a range of other factors (Bouchenak‐Khelladi *et al.*, 2015).

### Trait lability of polyploidy

The lack of strong phylogenetic signal for polyploidy in Pomaderreae suggests that ploidy as a trait is labile across this group (i.e. not phylogenetically constrained), similar to that of another Australasian plant group: Lomandroideae (Gunn *et al.*, 2020). This suggests that WGD, re-diploidization and high turnover of polyploids are common in Pomaderreae. Indeed, we found different ploidy levels even within species (e.g. *Pomaderris paniculosa* and *Cryptandra tomentosa s.l.*, both widely distributed, variable species; Supplementary Data Table S11). The lability of polyploids as a trait may explain why there are high numbers of triploids recorded in Pomaderreae, as ploidy levels are in flux, and we would expect these to transition either to diploid or tetraploid + (Ramsey and Schemske, 1998). Evolutionary lability has been associated with increases in diversification rates (Onstein, 2020; Singhal *et al.*, 2021); however, this was not the case for Pomaderreae. More broadly, it appears that angiosperms in general have a propensity to undergo repeated WGD, as is shown in numerous studies documenting many examples of WGD events throughout the angiosperm Tree of Life (De Bodt *et al.*, 2005; Tank *et al.*, 2015; Ren *et al.*, 2018). Cycles of polyploidization followed by re-diploidization have also been shown for numerous plant groups (Soltis *et al.*, 2016; Li *et al.*, 2021). Thus, trait lability of polyploidy as a proxy of frequent WGD in Pomaderreae is not surprising, as it fits the general pattern across angiosperms.

The lability of polyploidy may also be linked to biotic traits, as has been shown for Lomandroideae and their storage roots, as an enabler trait for diversification into novel biomes (Gunn *et al.*, 2020). Other studies have shown that floral traits generally increase in size following WGD events (Porturas *et al.*, 2019). Little is known about the ecology and evolutionary significance of different morphological traits for Pomaderreae. The presence of spines has been noted in Rhamnaceae genera such as *Paliurus*, *Colletia*, *Discaria*, *Cryptandra* and *Trymalium*. Spines may serve as defence against large herbivores, but for *Cryptandra*Nge *et al.* (2023) did not find significant differences in diversification rates between spinescent and non-spinescent lineages. Genera in Pomaderreae exhibit differences in leaf shape and size, with genera found in wetter climates (*Trymalium*, *Pomaderris*, *Spyridium*) usually having larger leaves and those in Mediterranean and arid regions (*Cryptandra*, *Stenanthemum*, *Blackallia*, *Papistylus*, *Serichonus*, *Polianthion*) having smaller leaves (Canning and Jessop, 1986; Walsh and Udovicic, 1999; Harden, 2000; Bean, 2004; Kellermann *et al.*, 2022*c*). *Cryptandra* species often have revolute leaves that become more pronounced during a dry season (Kellermann, 2020*a*). The presence of hairs (indumentum) particularly at the lower leaf surface is evident for almost all Pomaderreae taxa (Kellermann, 2020*a*). A wide variety of leaf indumentum forms have been documented for the tribe (Kellermann, 2020*a*); the function of these hairs is presumably related to the reflectance of excess solar radiation and reduction of transpiration – adaptations to drier climates. In terms of floral traits, Pomaderreae species exhibit a generalist insect pollination syndrome (Brundrett, 2021), with small white–yellow flowers. Some *Stenanthemum* and *Spyridium* species have floral leaves subtending the flowering inflorescences. Chen *et al.* (2023) surmised that Pomaderreae taxa have ant-dispersed seeds (myrmecochory) based on the presence of arils on the seeds. Eco-physiological traits such as stomata size and density have also been linked with polyploidy (and genome size) for other plant groups (Jordan *et al.*, 2015; van Mazijk *et al.*, 2024). However, little is known about these traits for Pomaderreae at present. Thus, gathering additional trait data to test for trait-dependent diversification in an explicit framework (Helmstetter *et al.*, 2023) linking it with polyploidy (Zenil-Ferguson *et al.*, 2017) and exploration of niche space (Bouchenak‐Khelladi *et al.*, 2015) for Pomaderreae would be especially promising.

### Outlook

Our study here focused on polyploidy of extant taxa and tip diversification rates to address questions on ploidy evolution, niche space exploration, and species richness patterns. The results presented here provide a framework for further studies on ploidy evolution and its impact on diversification. A promising next step would be to examine for ancient WGD events and linking that with ploidy and when these WGD events have occurred in the evolutionary history of Pomaderreae (and wider Rhamnaceae). It is possible that WGD events in deeper timescales have resulted in the diversification of certain clades, leading to more species-rich clades/genera such as *Pomaderris*, *Spyridium* and *Cryptandra.* It is well known that WGD events play an important role in the evolution and diversification of plants (Tank *et al.*, 2015; Clark and Donoghue, 2018; Landis *et al.*, 2018; Walden *et al.*, 2020). In addition, WGDs in angiosperms often coincide with major environmental events such as the K–Pg mass extinction (Vanneste *et al.*, 2014; Koenen *et al.*, 2021). However, transcriptome data would be required to investigate for WGD events and the precise timing of their occurrence over the course of evolutionary history for Pomaderreae.

Variation in genome size and its effects on diversification could also be another logical next step for research, though of course genome size is not always linked with polyploidy (Liu *et al.*, 2019; Moeglein *et al.*, 2020; Bhadra *et al.*, 2023). Our findings of extensive polyploidy across the Pomaderreae tribe and a significant positive relationship between polyploidy and genus size (species richness) suggest intrinsic biotic genetic drivers have resulted in present-day disparities in species richness across clades, space, and time.

## SUPPLEMENTARY DATA

Supplementary data are available at *Annals of Botany* online and consist of the following. Table S1: sequenced Pomaderreae taxa and associated herbarium voucher metadata included in this study. Table S2: summary statistics from distribution models, showing number of presence points, final list of climatic predictors, binarization threshold and resulting AUC for the modelling of each species. Table S3: the 30 orthologous nuclear loci used in this study. Table S4: ploidy estimates of 11 Pomaderris species from flow cytometry (Chen *et al.*, 2019) and nQuire based on sequence data from this study. Table S5: summary statistic of Spearman rank correlation tests between species richness for Pomaderreae genera and their ploidy frequency. *, ** and *** indicate significant *P* values of < 0.05, 0.01 and 0.001 respectively. Table S6: summary statistics of the Poisson regression analysis between genus size (total number of species per genus) and number of polyploid species (triploid and tetraploid +) per genus. *** indicates significant *P* values of < 0.001. Table S7: summary statistics of Fritz’s *D*-statistic test for phylogenetic signal of diploid versus polyploidy, based on 10 000 permutations. Table S8: summary statistics of Blomberg’s *K* and Pagel’s *λ* tests for phylogenetic signal of ploidy (diploid, triploid, tetraploid +). Table S9: summary of correlations between lineage age (Myr) and with polyploidy (3-PLOID and 2-PLOID) from phylANOVA analyses, accounting for phylogenetic relatedness. Table S10: ploidy estimates of non-Pomaderreae Rhamnaceae obtained from the KEW Plant DNA C-values database, and estimated using nQuire with newly sequenced data from this study. Table S11: ploidy estimates of multi-accessions of Pomaderreae species (*Cryptandra tomentosa s.l.* and *Pomaderris paniculosa*) using nQuire with newly sequenced data from this study. Figure S1: maximum likelihood RAxML phylogeny of Pomaderreae. Figure S2: species richness map derived from cleaned herbarium occurrence data of Pomaderreae across Australia excluding Pomaderris, using R packages sp and raster. Figure S3: boxplots showing (a) diversification rates, (b) speciation rates and (c) turnover rates for each ploidy level. Figure S4: boxplots showing the (a) range (max–min) and (b) midpoint values of elevation for each ploidy level. Figure S5: scaled PCA of niche breadth for Pomaderreae taxa in Australia based on different ploidy categories (red, diploid; blue, triploid, green, tetraploid +).

mcae181_suppl_Supplementary_Material
