# Supplementary material for: Polyploidy linked with species richness but not diversification rates or niche breadth in Australian Pomaderreae (Rhamnaceae)
Source: Ann Bot. 2024 Oct 23;135(3):531–48. doi: 10.1093/aob/mcae181 (PMC11920800; doi:10.1093/aob/mcae181)
Supplement: mcae181_suppl_Supplementary_Material [file mcae181_suppl_supplementary_material.zip › mcae181_suppl_supplementary_material.docx]

Supplementary Materials

**Polyploidy linked with species richness but not diversification rates or niche breath in Australasian Pomaderreae**

Francis J. Nge^1,2,3,5^, Timothy A. Hammer^2,3^, Thais Vasconcelos^4^, Ed Biffin^2,3^, Juergen Kellermann^2,3^, Michelle Waycott^2,3^

Supplementary Methods and Results

**Table S1.** Sequenced Pomaderreae taxa and associated herbarium voucher metadata included in this study (included in separate csv file).

**Table S2.** Summary statistics from distribution models, showing number of presence points, final list of climatic predictors, binarization threshold and resulting AUC for the modelling of each species (included in separate csv file).

**Table S3.** The thirty orthologous nuclear loci used in this study.

| contig | *Arabidopsis thaliana* | gene name | NCBI Reference sequence (position on refseq) |
| --- | --- | --- | --- |
| contig 5 | AT1G49540.2 | elongator protein 2 | XM_016021267.2 (748..1,943) |
| contig 121 | AT3G01660.1 | S-adenosyl-L-methionine-dependent methyltransferases superfamily protein | XM_016023528.2 (136..1,048) |
| contig 17 | AT2G38270.1 | CAX-interacting protein 2 | XM_016041750.2 (469..1,008) |
| contig 171 | AT2G22650.1 | FAD-dependent oxidoreductase family protein | XM_016037427.2 (975..1,430) |
| contig 179 | AT2G40570.1 | initiator tRNA phosphoribosyl transferase family protein | XM_016035496.2 (70..186) |
| contig 186 | AT1G77930.1 | Chaperone DnaJ-domain superfamily protein | XM_025076062.1 (141..477) |
| contig 208 | AT5G39410.1 | Saccharopine dehydrogenase | XM_021602434.2 (395..952) |
| contig 229 | AT1G12370.2 | photolyase 1 | XM_016041072.2 (272..1,103) |
| contig 25 | AT1G49380.1 | cytochrome c biogenesis protein family | XM_016047385.2 (552..757) |
| contig 26 | AT1G05055.1 | general transcription factor II H2 | XM_031106615.1 (713..1,303) *Quercus lobata* |
| contig 27 | AT5G56740 | histone acetyltransferase of the GNAT family 2 | XM_025076092.1 (902..1,016) |
| contig 282 | AT1G07970.1 | PTHR21780:SF0 - TRANSMEMBRANE PROTEIN 209 | XM_016022952.2 (764..1,976) |
| contig 301 | AT3G15290.1 | 3-hydroxyacyl-CoA dehydrogenase family protein | XM_016031844.2 (677..793) |
| contig 340 | AT4G03200.1 | PTHR12145:SF11 - SPERMATOGENESIS-ASSOCIATED PROTEIN 20 | XM_025077671.1 (1,194..1,306) |
| contig 356 | AT3G09180.1 | K15170 - mediator of RNA polymerase II transcription subunit 27 (MED27) | XM_025067131.1 (287..750) |
| contig 377 | AT3G17040.1 | high chlorophyll fluorescent 107 | XM_025073955.1 (1,471..1,581) |
| contig 378 | AT5G65860.1 | ankyrin repeat family protein | XM_016029545.2 (817..1,145) |
| contig 459 | AT2G31840.1 | Thioredoxin superfamily protein | XM_016024034.2 (371..1,007) |
| contig 498 | AT1G15390.1 | peptide deformylase 1A | XM_016015676.2 (620..1,012) |
| contig 585 | AT1G21370.1 | KOG4533 - Uncharacterized conserved protein | XM_016030909.2 (1,000..1,371) |
| contig 606 | AT3G26580.1 | Tetratricopeptide repeat (TPR)-like superfamily protein | XM_016039159.2 (586..717) |
| contig 61 | AT4G36390.1 | Methylthiotransferase | XM_016034390.2 (407..2,135) |
| contig 62 | AT4G26980.1 | RNI-like superfamily protein | XM_016044316.2 (820..1,247) |
| contig 66 | AT4G33030.1 | sulfoquinovosyldiacylglycerol 1 | XM_025074823.1 (722..1,278) |
| contig 68 | AT1G74640.1 | alpha/beta-Hydrolases superfamily protein | XM_016012577.2 (832..1,380) |
| contig 714 | AT5G10920.1 | L-Aspartase-like family protein | XM_016030483.2 (855..1,005) |
| contig 773 | AT4G35910.1 | Adenine nucleotide alpha hydrolases-like superfamily protein | XM_016042688.2 (727..816) |
| contig 78 | AT2G04560.1 | transferases, transferring glycosyl groups | XM_016026012.2 (587..1,136) |
| contig 87 | AT5G64150.1 | RNA methyltransferase family protein | XM_025075431.1 (233..1,113) |
| contig 98 | AT2G02590.1 | PF06695 - Putative small multi-drug export protein (Sm_multidrug_ex) | XM_016039395.2 (986..1,579) |

**Table S4.** Ploidy estimates of 11 *Pomaderris* species from flow cytometry (Chen *et al.* 2019) and nQuire based on sequence data from this study. Taxa with conflicting ploidy estimates are indicated in bold and ‘*’. Number of reads sequenced per sample with colours going from red–orange–yellow–green (low–high).

|  | **From Chen et al. 2019** | | | **nQuire** |  |  |
| --- | --- | --- | --- | --- | --- | --- |
| **Taxa** | **Genome.size (2C) pg** | **Genome size (1C)** | **Ploidy** | **Ploidy** | **number of reads (sequence)** | **Same sample as Chen et al. 2019** |
| *Pomaderris bodalla* | 0.97 | 0.48 | **2** | **3*** | 2348234 | Yes: *J. McAuliffe 1955* |
| *Pomaderris brunnea* | 0.95 | 0.47 | 2 | 2 | 710522 | Yes: *J.L. Percival 120* |
| *Pomaderris cinerea* | 0.86 | 0.43 | 2 | 2 | 1639742 | NO: our sample (*J. McAuliffe 1767*), Chen (*J. McAuliffe 1766*) |
| *Pomaderris cotoneaster* | 1.86 | 0.93 | 4 | 4 | 1383302 | Yes: *J. McAuliffe 1805* |
| *Pomaderris delicata* | 0.91 | 0.45 | 2 | 2 | 2771184 | Yes: *J. McAuliffe 1966* |
| *Pomaderris obcordata* | 0.88 | 0.44 | **2** | **3*** | 612214 | NO |
| *Pomaderris pallida* | 1.39 | 0.70 | 3 | 3 | 1604222 | Yes: *M.L. Henery 44* |
| *Pomaderris parrisiae* | 0.78 | 0.39 | **2 or 4** | **4*** | 1070544 | Yes: *F. Nge 385* |
| *Pomaderris velutina* | 0.89 | 0.45 | 2 | 2 | 7031372 | NO: our sample (*J. McAuliffe 1754*); Chen (*J. McAullifee 1753*) |
| *Pomaderris walshii* | 1.99 | 1.00 | 4 | 4 | 14557204 | Yes: *S. Pedessen 1407* |
| *Pomaderris forrestiana* | 0.99 | 0.49 | 2 | 2 | 6425884 | NO: our sample (*C. Clowes 554*);  Chen (*D.J. Duval 1530* [AD]) |

**Table S5.** Summary statistic of Spearman rank correlation tests between species richness for Pomaderreae genera and their ploidy frequency. *, **, *** indicate significant *p* values of 0.05, 0.01, and 0.001 respectively.

| **Spearman test** | **rho** | **p value** |
| --- | --- | --- |
| log species ~ diploid percentage | -0.885 | <0.001*** |
| log species ~ triploid percentage | 0.855 | <0.01** |
| log species ~ tetraploid+ percentage | 0.687 | <0.05* |

**Table S6.** Summary statistic of the Poisson regression analysis between genus size (total number of species per genus) and number of polyploid species (triploid and tetraploid+) per genus. *** indicate significant *p* values of 0.001. The null deviance value is 316 (9 degrees of freedom; df), residual deviance is 86.37 (8 df), and AIC score value of 129.78.

| **Variable** | **Estimate** | **Std. Error** | **z Value** | ***p* value** |
| --- | --- | --- | --- | --- |
| Intercept | 2.12 | 0.123 | 17.18 | <2e-16 *** |
| meta$no.poly | 0.057 | 0.004 | 14.59 | <2e-16 *** |

**Table S7.** Summary statistic of Fritz’s *D-*statistic test for phylogenetic signal of diploid vs. polyploidy, based on 10,000 permutations.

| **Test** | ***p* value** |
| --- | --- |
| Probability of E(D) resulting from no (random) phylogenetic structure | <0.0001 |
| Probability of E(D) resulting from Brownian phylogenetic structure | 0.003 |

**Table S8.** Summary statistic of Blomberg’s K and Pagel’s lambda tests for phylogenetic signal of ploidy (diploid, triploid, tetraploid+). Non-significant *p* values indicate no phylogenetic signal. Blomberg’s K *p* value was derived based on 1000 simulations.

| **Test** | **Phylogenetic signal** | ***p* value** | **logL (lambda)** | **LR (lambda=0)** |
| --- | --- | --- | --- | --- |
| Blomberg's K | 0.24 | 0.092 | na | na |
| Pagel’s λ | 0.0497 | 0.099 | -231.5 | 2.719 |

**Table S9.** Summary of correlations between lineage age (Myr) and with polyploidy (3-PLOID and 2-PLOID) from phylANOVA analyses, accounting for phylogenetic relatedness.

|  | **3-PLOID (diploid, triploid, tetraploid+)** | | | **2-PLOID (diploid, polyploid)** | | |
| --- | --- | --- | --- | --- | --- | --- |
| **Variable** | **Sum sq** | **Residual** | ***p-*value** | **Sum sq** | **Residual** | ***p-*value** |
| Lineage age | 0.01 | 0.67 | 0.68 | 89.42 | 2084.14 | 0.246 |

**Table S10.** Ploidy estimates of non-Pomaderreae Rhamnaceae obtained from the KEW Plant DNA C-values database, and estimated using nQuire with newly sequenced data from this study. Taxa that are estimated to be polyploids based on this study are highlighted in green.

| **Genus** | **Species** | **DNA Amount 1C (pg)** | **Averaging**  **0.25** | **nQuire** | **Original Reference** |
| --- | --- | --- | --- | --- | --- |
| *Rhamnus* | *alpinus* | 0.25 |  |  | Siljak-Yakovlev *et al.* 2010 |
| *Rhamnus* | *alnifolia* | 0.29 |  |  | Fridley & Craddock 2015 |
| *Rhamnus* | *alaternus* | 0.31 |  |  | Siljak-Yakovlev *et al.* 2010 |
| *Paliurus* | *spina-christi* | 0.33 | 1.3 |  | Siljak-Yakovlev *et al.* 2010 |
| *Frangula* | *alnus* | 0.33 | 1.3 |  | Siljak-Yakovlev *et al.* 2010 |
| *Frangula* | *rupestris* | 0.34 | 1.4 |  | Siljak-Yakovlev *et al.* 2010 |
| *Rhamnus* | *frangula* | 0.34 | 1.4 | 3.0 | Sonnier,2016 |
| *Frangula* | *caroliniana* | 0.44 | 1.8 | 4.0 | Fridley & Craddock 2015 |
| *Rhamnus* | *intermedius* | 0.45 | 1.8 |  | Siljak-Yakovlev *et al.* 2010 |
| *Rhamnus* | *orbiculata* | 0.47 | 1.9 |  | Siljak-Yakovlev *et al.* 2010 |
| *Rhamnus* | *davurica* | 0.53 | 2.1 |  | Fridley & Craddock 2015 |
| *Ceanothus* | *americanus* | 0.55 | 2.2 |  | Bai *et al.* 2012 |
| *Rhamnus* | *saxatilis* | 0.56 | 2.2 |  | Siljak-Yakovlev *et al.* 2010 |
| *Rhamnus* | *cathartica* | 0.58 | 2.3 | 4.0 | Pustahija *et al.* 2013 |
| *Ceanothus* | *herbaceus* | 0.59 | 2.4 |  | Sonnier 2016 |
| *Colubrina* | *asiatica* | 0.93 | 3.7 | 4.0 | Ohri *et al.* 2004 |
| *Ziziphus* | *glabrata* | 1.55 | 6.2 |  | Ohri 2002 |

**Table S11.** Ploidy estimates of multi-accessions of Pomaderreae species (*Cryptandra tomentosa s.l.* and *Pomaderris paniculosa*) using nQuire with newly sequenced data from this study.

| **Taxa** | **Sequence_name** | **HC** | **PLOIDY** |
| --- | --- | --- | --- |
| *Cryptandra_tomentosa_s.l.* | Cryptandra_tomentosa_FN_1262 | HC 73 | 2 |
| *Cryptandra_tomentosa_s.l.* | Cryptandra_tomentosa_Monarto_JK_649 | HC 69 | 2 |
| *Cryptandra_tomentosa_s.l.* | Cryptandra_tomentosa_sp_Floriferous_Yacka_Cemetery_Reserve_FN_977 | HC 69 | 4 |
| *Cryptandra_tomentosa_s.l.* | Cryptandra_tomentosa_spinose_JK_779 | HC 73 | 2 |
| *Cryptandra_tomentosa_s.l.* | Cryptandra_tomentosa_spinose_JK_855 | HC 73 | 3 |
| *Cryptandra_tomentosa_s.l.* | Cryptandra_tomentosa_spinose_JK_894 | HC 73 | 3 |
| *Cryptandra_tomentosa_s.l.* | Cryptandra_tomentosa_spinose_JK_895 | HC 73 | 2 |
| *Cryptandra_tomentosa_s.l.* | Cryptandra_tomentosa_spiny_hairy_flowers_all_over_Murray_Stockyard_Plain_SA_FN_975 | HC 69 | 2 |
| *Cryptandra_tomentosa_s.l.* | Cryptandra_tomentosa_spiny_W_Eyre_peninsula_NNW_of_Arno_Bay_FN_979 | HC 69 | 2 |
| *Cryptandra_tomentosa_s.l.* | Cryptandra_tomentosa_tomentosa_spiny_hairy_sepals_tips_only_Murray_FN_981 | HC 69 | 2 |
| *Cryptandra_tomentosa_s.l.* | Cryptandra_tomentosa_typical_JK_659 | HC 39 | 3 |
| *Cryptandra_tomentosa_s.l.* | Cryptandra_tomentosa_typical_JK_746 | HC 39 | 2 |
| *Cryptandra_tomentosa_s.l.* | Cryptandra_tomentosa_typical_JK_810_2 | HC 73 | 2 |
| *Cryptandra_tomentosa_s.l.* | Cryptandra_tomentosa_typical_JK_933 | HC 73 | 2 |
| *Cryptandra_tomentosa_s.l.* | Cryptandra_tomentosa_typical_JK_944 | HC 73 | 3 |
| *Cryptandra_tomentosa_s.l.* | Cryptandra_tomentosa_var_floribunda_Big_Desert,_Victoria_FN_973 | HC 69 | 4 |
| *Cryptandra_tomentosa_s.l.* | Cryptandra_tomentosa_var_floribunda_FN_1260 | HC 73 | 3 |
| *Cryptandra_tomentosa_s.l.* | Cryptandra_tomentosa_var_floribunda_Portion_90_Wyberba_Granite_dome_FN_971 | HC 69 | 2 |
| *Cryptandra_tomentosa_s.l.* | Cryptandra_tomentosa_var_floribunda_The_Springs,_31_km_E_of_Ashford_FN_972 | HC 69 | 2 |
| *Cryptandra_tomentosa_s.l.* | Cryptandra_tomentosa_var_floribunda_Wanilla_Wanilla,_Eyre_peninsula_JK_746 | HC 69 | 2 |
|  |  |  |  |
| *Pomaderris_paniculosa* | Rham_232_Pomaderris_paniculosa_FN_289_2_WA_trimmed_(paired)_ | HC 42 | 3 |
| *Pomaderris_paniculosa* | Rham_233_Pomaderris_paniculosa_FN_289_3_WA_trimmed_(paired)_ | HC 42 | 3 |
| *Pomaderris_paniculosa* | Rham_234_Pomaderris_paniculosa_FN_289_4_WA_trimmed_(paired)_ | HC 42 | 3 |
| *Pomaderris_paniculosa* | Rham_235_Pomaderris_paniculosa_FN_201_2_SA_army_trimmed_(paired)_ | HC 42 | 2 |
| *Pomaderris_paniculosa* | Rham_237_Pomaderris_paniculosa_FN_201_4_SA_army_trimmed_(paired)_ | HC 42 | 2 |
| *Pomaderris_paniculosa* | Rham_238_Pomaderris_paniculosa_FN_204_1_SA_Murray_trimmed_(paired)_ | HC 42 | 2 |
| *Pomaderris_paniculosa* | Rham_239_Pomaderris_paniculosa_FN_204_3_SA_Murray_trimmed_(paired)_ | HC 42 | 2 |
| *Pomaderris_paniculosa* | Rham_240_Pomaderris_paniculosa_FN_204_4_SA_Murray_trimmed_(paired)_ | HC 42 | 2 |
| *Pomaderris_paniculosa* | Rham_241_Pomaderris_paniculosa_subsp_paniculosa_FN_931_trimmed_(paired)_ | HC 42 | 2 |
| *Pomaderris_paniculosa* | Rham_242_Pomaderris_paniculosa_subsp_paniculosa_FN_933_trimmed_(paired)_ | HC 42 | 2 |
| *Pomaderris_paniculosa* | Rham_243_Pomaderris_paniculosa_subsp_paniculosa_FN_935_trimmed_(paired)_ | HC 42 | 4 |
| *Pomaderris_paniculosa* | Rham_246_Pomaderris_paniculosa_subsp_paniculosa_AD_183300_FN_940_trimmed_(paired)_ | HC 42 | 2 |
| *Pomaderris_paniculosa* | Rham_248_Pomaderris_paniculosa_subsp_paniculosa_AD_240132_FN_943_trimmed_(paired)_ | HC 42 | 2 |
| *Pomaderris_paniculosa* | Rham_250_Pomaderris_paniculosa_subsp_paniculosa_FN_946_trimmed_(paired)_ | HC 42 | 3 |
| *Pomaderris_paniculosa* | Rham_251_Pomaderris_paniculosa_subsp_paniculosa_FN_947_trimmed_(paired)_ | HC 42 | 3 |
| *Pomaderris_paniculosa* | Rham_368_Pomaderris_paniculosa_SA_FN_782_4 | HC 70 | 3 |
| *Pomaderris_paniculosa* | Rham_370_Pomaderris_paniculosa_SA_FN_756_4 | HC 70 | 4 |
| *Pomaderris_paniculosa* | Rham_371_Pomaderris_paniculosa_SA_FN_756_5 | HC 70 | 3 |
| *Pomaderris_paniculosa* | Rham_372_Pomaderris_paniculosa_SA_FN_201_3 | HC 70 | 3 |
| *Pomaderris_paniculosa* | Rham_375_Pomaderris_paniculosa_SA_FN_1030_2 | HC 70 | 4 |
| *Pomaderris_paniculosa* | Rham_376_Pomaderris_paniculosa_SA_FN_1030_3 | HC 70 | 4 |
| *Pomaderris_paniculosa* | Rham_377_Pomaderris_paniculosa_SA_FN_1030_4 | HC 70 | 4 |
| *Pomaderris_paniculosa* | Rham_378_Pomaderris_paniculosa_SA_FN_1030_5 | HC 70 | 4 |
| *Pomaderris_paniculosa* | Rham_387_Pomaderris_paniculosa_SA_KI_FN_1189_2 | HC 70 | 4 |
| *Pomaderris_paniculosa* | Rham_396_Pomaderris_paniculosa_subsp_paralia_Vic_FN_1146_1 | HC 70 | 3 |
| *Pomaderris_paniculosa* | Rham_398_Pomaderris_paniculosa_subsp_paralia_Vic_FN_1146_3 | HC 70 | 3 |
| *Pomaderris_paniculosa* | Rham_399_Pomaderris_paniculosa_subsp_paralia_Vic_FN_1146_4 | HC 70 | 4 |
| *Pomaderris_paniculosa* | Rham_401_Pomaderris_paniculosa_subsp_paralia_Vic_FN_1149_1 | HC 70 | 3 |
| *Pomaderris_paniculosa* | Rham_403_Pomaderris_paniculosa_subsp_paralia_Vic_FN_1149_3 | HC 70 | 3 |

**Fig. S1a.** Maximum likelihood RAxML phylogeny of Pomaderreae. Node support are bootstrap support values (attach as separate pdf file).

**Fig. S1b.** Maximum likelihood phylogeny of Pomaderreae with diploid-only taxa, inferred from nQuire. Node support are bootstrap support values (attach as separate pdf file).

**Fig. S2**. Species richness map derived from cleaned herbarium occurrence data of Pomaderreae across Australia excluding *Pomaderris,* using R packages sp and raster*.* Left y-axis and x-axes respectively show latitude and longitude coordinates. Right y-axis indicate number of species (high–low; red–blue).

**Fig. S3.** Boxplots showing (a) diversification rates, (b) speciation rates, and (c) turnover rates for each ploidy level. Boxes represent the interquartile range with the central horizontal bar in each box showing the median, and white dot showing the mean. Whiskers show location of most extreme data points that are still within a factor of 1.5 of the upper and lower quartiles, and black points show outliers outside this range. Bars over boxplots indicate significant differences between different regions, with * indicating a significant *p* value of < 0.05.

**Fig. S3a.** log net tip diversification rates.

**Fig. S3b.** log speciation rates.

**Fig. S3c.** log turnover rates.

**Fig. S4.** Boxplots showing the (a) range (max-min), and (b) midpoint values of elevation for each ploidy level. Boxes represent the interquartile range with the central horizontal bar in each box showing the median, and white dot showing the mean. Whiskers show location of most extreme data points that are still within a factor of 1.5 of the upper and lower quartiles, and black points show outliers outside this range. Bars over boxplots indicate significant differences between different regions, with * indicating a significant *p* value of < 0.05.

**Fig. S4a.** log elevation range values.

**Fig. S4b.** log elevation midpoint values.

**Fig. S5**. Scaled principal component analysis of niche breadth for Pomaderreae taxa in Australia based on different ploidy categories (red – diploid, blue – triploid, green – tetraploid+). Niche breadth is based on log transformed WorldClim Bio1–19 variables.

Outlines indicate the 95% confidence ellipses for each ploidy category.

**Supplementary literature cited:**

(Siljak-Yakovlev et al., 2010, Fridley and Craddock, 2015, Bai et al., 2012, Pustahija et al., 2013, Ohri et al., 2004)

**Bai C, Alverson WS, Follansbee A, Waller DM.** **2012**. New reports of nuclear DNA content for 407 vascular plant taxa from the United States. *Annals of Botany,* **110**: 1623–1629.

**Fridley JD, Craddock A.** **2015**. Contrasting growth phenology of native and invasive forest shrubs mediated by genome size. *New Phytologist,* **207**: 659–668.

**Ohri D, Bhargava A, Chatterjee A.** **2004**. Nuclear DNA Amounts in 112 Species of Tropical Hardwoods‐New Estimates. *Plant Biology,* **6**: 555–561.

**Pustahija F, Brown SC, Bogunić F, Bašić N, Muratović E, Ollier S, Hidalgo O, Bourge M, Stevanović V, Siljak-Yakovlev S.** **2013**. Small genomes dominate in plants growing on serpentine soils in West Balkans, an exhaustive study of 8 habitats covering 308 taxa. *Plant and Soil,* **373**: 427–453.

**Siljak-Yakovlev S, Pustahija F, Šolić E, Bogunić F, Muratović E, Bašić N, Catrice O, Brown S.** **2010**. Towards a genome size and chromosome number database of Balkan flora: C-values in 343 taxa with novel values for 242. *Advanced Science Letters,* **3**: 190–213.
